# Feasibility of Manufacturing and Antitumor Activity of TIL for Advanced Endometrial Cancers

**DOI:** 10.3390/ijms26157151

**Published:** 2025-07-24

**Authors:** Yongliang Zhang, Kathleen N. Moore, Amir A. Jazaeri, Judy Fang, Ilabahen Patel, Andrew Yuhas, Patrick Innamarato, Nathan Gilbert, Joseph W. Dean, Behzad Damirchi, Joe Yglesias, Rongsu Qi, Michelle R. Simpson-Abelson, Erwin Cammaart, Sean R. R. Hall, Hequn Yin

**Affiliations:** 1 Iovance Biotherapeutics, Inc., San Carlos, CA 94070, USA or yoz4.hz@gmail.com (Y.Z.); judy.fang@iovance.com (J.F.); ilabahen.patel@iovance.com (I.P.); andrew.yuhas@iovance.com (A.Y.); patrick.innamarato@iovance.com (P.I.); nathan.gilbert@iovance.com (N.G.); joe.dean@iovance.com (J.W.D.); behzad.damirchi@iovance.com (B.D.); joe.yglesias@iovance.com (J.Y.); rongsu.qi@iovance.com (R.Q.); michelle.abelson@iovance.com (M.R.S.-A.); erwin.cammaart@iovance.com (E.C.); hequn.yin@iovance.com (H.Y.); 2 Stephenson Cancer Center, University of Oklahoma Health Sciences Center, Oklahoma City, OK 73104, USA; kathleen-moore@ouhsc.edu; 3 University of Texas MD Anderson Cancer Center, Houston, TX 77030, USA; aajazaeri@mdanderson.org

**Keywords:** immunotherapy, uterine neoplasms, mismatch repair, tumor-infiltrating lymphocytes

## Abstract

Lifileucel, a tumor-infiltrating lymphocyte (TIL) cell therapy approved for advanced melanoma, demonstrates promise for treating other solid tumors, including endometrial cancer (EC). The current study evaluates the feasibility of manufacturing TILs from EC tumors using Iovance’s proprietary 22-day Gen2 manufacturing process. Key parameters, including TIL yield, viability, immune phenotype, T-cell receptor clonality, and cytotoxic activity, were assessed. Of the 11 EC tumor samples processed at research scale, 10 (91%) successfully generated >1 × 10^9^ viable TIL cells, with a median yield of 1.1 × 10^10^ cells and a median viability of 82.8%. Of the four EC tumor samples processed at full scale, all achieved the pre-specified TVC and viability targets. Putative tumor-reactive T-cell clones were maintained throughout the manufacturing process. Functional reactivity was evidenced by the upregulation of 4-1BB in CD8+ T cells, OX40 in CD4+ T cells, and increased production of IFN-γ and TNF-α upon autologous tumor stimulation. Furthermore, antitumor activity was confirmed using an in vitro autologous tumor organoid killing assay. These findings demonstrate the feasibility of ex vivo TIL expansion from EC tumors. This study provides a rationale for the initiation of the phase II clinical trial IOV-END-201 (NCT06481592) to evaluate lifileucel in patients with advanced EC.

## 1. Introduction

Endometrial cancer (EC) is the most common gynecologic malignancy in developed countries, with rising incidence rates globally [1]. While early-stage EC is often curable with surgery and adjuvant therapies, advanced or recurrent disease remains a significant clinical challenge, with limited treatment options and poor survival outcomes [1]. Immune checkpoint inhibitors (ICIs) have shown promise in a subset of patients with EC, particularly those with mismatch repair-deficient (dMMR) or microsatellite instability-high (MSI-H) tumors [2]. However, even in this subgroup, at least 20% of patients experience recurrence within 12 months and others may experience recurrences later [2]. Furthermore, among patients with the more common proficient MMR (pMMR) tumors, the addition of ICI to chemotherapy and following chemotherapy has modestly improved progression-free survival, but there is no sign of overall survival benefit [3,4]. Both of these clinical scenarios point to the need for continued therapeutic development in this space.

Tumor-infiltrating lymphocytes (TILs) represent a promising avenue for adoptive cell therapy (ACT) in solid tumors [5,6]. TILs are naturally occurring T cells that infiltrate tumors and possess intrinsic tumor-specific reactivity. Preclinical and clinical studies have demonstrated that TILs can recognize and eliminate tumor cells through diverse mechanisms, including the release of cytotoxic molecules and cytokines, as well as direct cell-to-cell contact [7,8,9]. The success of TIL therapy in advanced melanoma, exemplified by the recent FDA approval of lifileucel [10], has sparked interest in extending this approach to other solid tumors, including EC. However, the feasibility of generating TILs from EC tumors and their functional characterization remain underexplored.

The manufacturing of TILs for therapeutic use involves the isolation, ex vivo expansion, and reinfusion of autologous TILs into patients. Iovance’s proprietary 22-day Generation 2 (Gen2) manufacturing process has been implemented to produce large quantities of viable, tumor-reactive TILs while maintaining their functional potency [11,12]. This process has been applied in melanoma and other solid tumors, including NSCLC, head and neck cancer, and cervical cancer. Key considerations in TIL therapy for EC include the heterogeneity of the tumor microenvironment, the variability in TIL infiltration across tumor subtypes, and the need to preserve tumor-reactive T-cell clones during expansion [13,14].

The current study aims to evaluate the feasibility of manufacturing TILs from EC tumors using the Gen2 process and to characterize their phenotypic and functional properties. Critical parameters were assessed, including TIL yield, viability, immune phenotype, and T-cell receptor (TCR) clonality. Additionally, the tumor-reactive activity of TILs was investigated through activation marker expression and cytokine production. Through addressing the challenges of TIL manufacturing and demonstrating robust antitumor activity, this study lays the groundwork for advancing TIL therapy as a viable treatment option for EC patients.

## 2. Results

### 2.1. Successful Manufacturing of Endometrial TIL

To study the feasibility of manufacturing endometrial TILs, 11 distinct patients’ EC tumor samples, comprising five dMMR and six pMMR cases (Table A1), were processed. The tumor samples were from patients with EC aged 45–84 years, predominantly White (*n* = 10), with one Hispanic patient. Histopathology revealed endometrioid adenocarcinoma/carcinoma in 10 cases with FIGO grading ranging from I to III, with 73% (8/11) classified as grade II and one case as high-grade serous carcinoma (Table A1).

The tumor manufacturing outcomes (Table 1) demonstrated robust viability and scalability: 10 of 11 samples (91%) yielded >1 × 10^9^ extrapolated total viable cells (TVC), with a median TVC rate of 1.1 × 10^10^ and a median viability of 82.8%. High CD45+CD3+ T cells were enriched in the final TIL product (median: 97%, range: 90.2–98.3%). Notably, CD8+ T-cell proportions varied significantly across samples (6.4–63.1%), with the highest CD8+ infiltration (63.1%) observed in END22098 (the Hispanic patient’s tumor). Conversely, CD4+ T-cell dominance (>75%) characterized four cases. Only one sample (END22063) failed viability testing, highlighting occasional processing challenges. The data also indicate that manufacturing can be successfully achieved regardless of MMR status (Table 1 and Table A1). These results underscore the feasibility of generating TIL-based immunotherapies from EC tumors.

### 2.2. Phenotypic Characterization of Endometrial TILs

**Figure 1:**
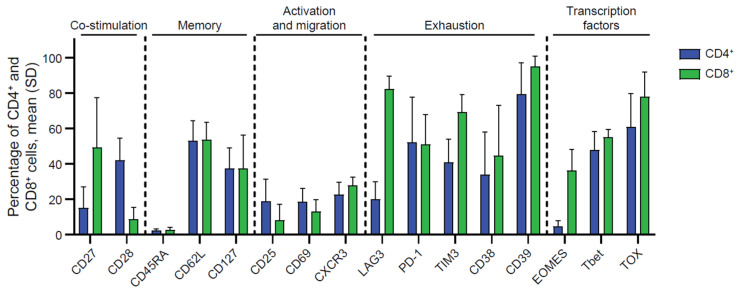
Phenotypes of endometrial TIL were characterized by flow cytometry. Cellular phenotypic analysis expression markers related to co-stimulation, memory, activation, migration, exhaustion, and transcription factors in both CD4+ T and CD8+ T subsets. Data generated from EC TIL samples were presented as expression frequency (*n* = 5, biological donors). See Figure S1 for gating strategy.

Flow cytometry analyses were performed to characterize TIL phenotype. As shown in Figure 1, the data revealed immune profiles consistent with historical data of TILs derived from other solid tumor types [15]. Analysis of five EC-derived TILs revealed that both the CD4+ and CD8+ subsets expressed markers associated with co-stimulation (CD27 and CD28), memory (CD45RA, CD62L, CD127), activation (CD25 and CD69), migration (CXCR3), exhaustion (LAG3, PD-1, TIM3, CD38, CD39), and transcription factors known to regulate these molecules and effector functions (EOMES, Tbet, and TOX). Notably, CD8+ TILs, in comparison to CD4+ TILs, had different expressions of various markers, including increased CD27, LAG3, TIM3, and EOMES. The expression of these markers in conjunction with Tbet, TOX, memory markers, and exhaustion markers are suggestive of an effector memory, T progenitor exhausted, or terminal exhaustion phenotype, all of which are critical for antitumor immunity [16,17].

### 2.3. Single-Cell Analysis of Endometrial TILs

**Figure 2:**
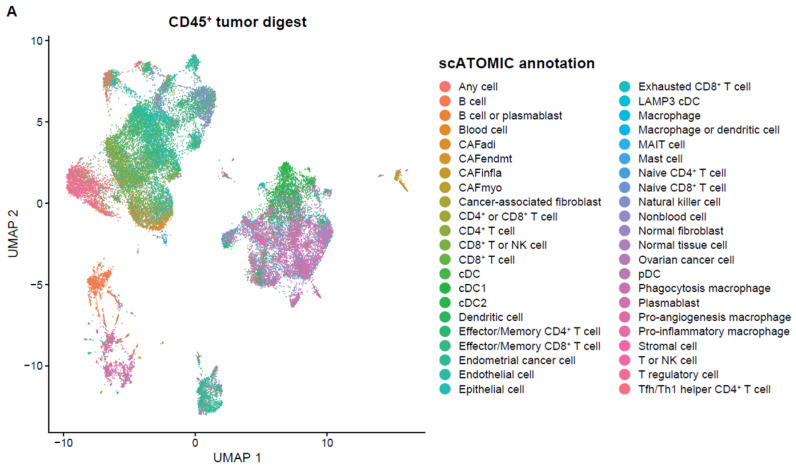

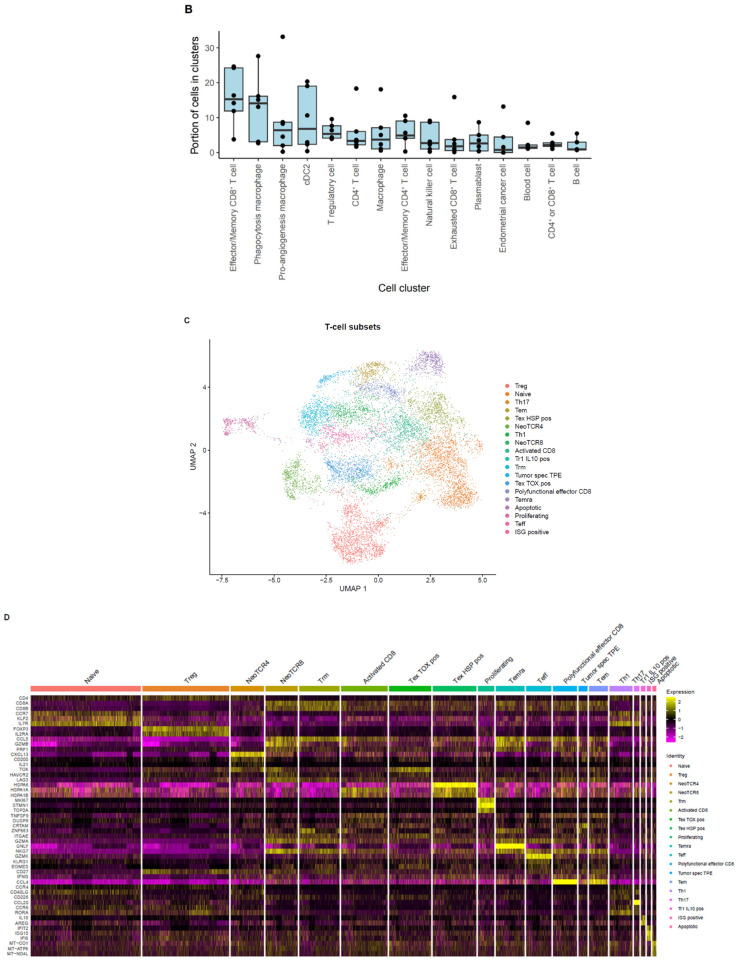

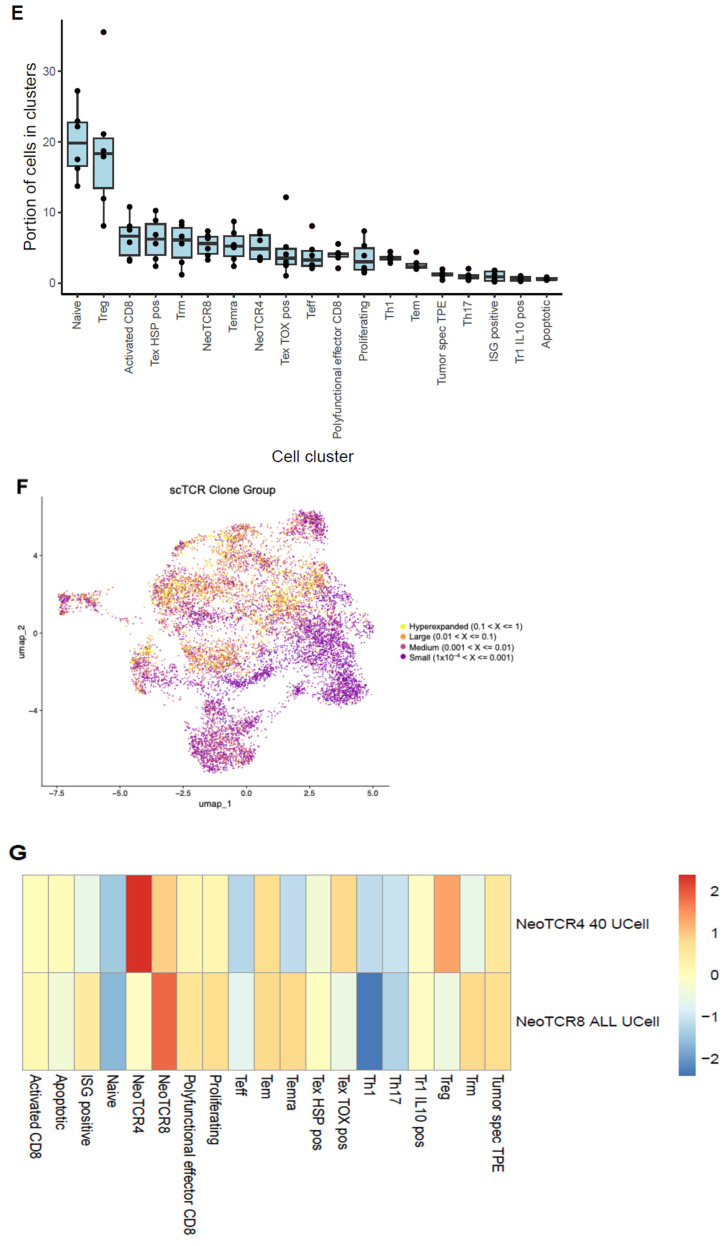

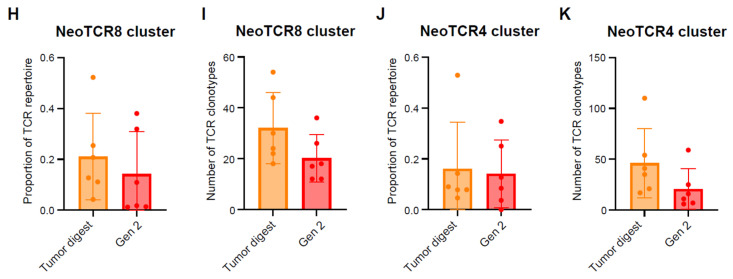
Single cell analysis of endometrial tumor infiltrating leukocytes and tracking of putative neoantigen-reactive T cells. (**A**) UMAP dimensionality reduction visualization projecting cell type annotations using the scAtomic package (v2.0.2) on integrated single-cell data consisting of 6 EC CD45+ tumor samples (**B**) Proportion of cells in the top 15 cell types ordered based on the average value across the 6 EC CD45+ tumor samples. (**C**) Integrated single-cell T-cell subset with 19 manually annotated cell types. (**D**) Heatmap of the normalized gene expression for the top marker genes detected in each cell type. (**E**) Proportion of cells in the T cell integrated single-cell data ordered based on the average value across 6 samples. (**F**) Integrated T-cell subset UMAP dimensionality reduction visualization projecting single cell TCR clone group, labeled based on the frequency of clones (X): hyperexpanded (0.1 < X < 1), large (0.01 < X < 0.1), medium (0.001 < X < 0.01) and small (1 × 10^−4^ < X < 0.001). (**G**) Expression level of the NeoTCR8 and NeoTCR4 gene set scores for all clusters. Clusters with the highest score for the NeoTCR8 and NeoTCR4 gene sets were annotated as NeoTCR8 and NeoTCR4 cell clusters, respectively. TCR clonotypes from the NeoTCR8 cluster were identified and tracked from tumor digest to TIL drug product via bulk TCR-seq. The clonotype proportion (**H**) and number (**I**) of unique clonotypes for each sample are summarized. TCR clonotypes from the NeoTCR4 cluster were identified and tracked from tumor digest to Gen2 TIL drug product via bulk TCR-seq. The clonotype proportion (**J**) and number (**K**) of unique clonotypes for each sample are summarized. Six biological replicates were used for the single-cell analysis.

With the end goal of assessing the neoantigen-reactive T-cell population of EC tumors, single cell RNA+TCR sequencing was performed on six CD45+ enriched EC tumor digest samples. After QC and filtering, a total of 37,054 single cells remained for downstream analysis (Table A2). Using a pan-cancer automated cell type annotation tool, a notable proportion of cells were annotated as macrophage, cDC2, and T-regulatory (Treg) cells (Figure 2A,B), suggesting an immunosuppressive tumor microenvironment consistent with previous reports for EC [18]. T cells were then subgrouped and manual annotation of clusters resulted in 19 unique tumor-infiltrating T-cell subsets (Figure 2C,D). Naive/memory-like (marked by CCR7, KLF2, and IL7R) and Treg (marked by FOXP3 and IL2RA) were among the most prevalent cell types among T cells (Figure 2D,E). Interestingly, these two populations mainly expressed TCRs that fell into the small- or medium-proportion scTCR clone group, whereas clusters of CD8+ T cells that exhibited an exhausted, progenitor exhausted, activated, or effector-like phenotype appeared to express the higher frequency (Hyperexpanded) TCR clones found in the tumor (Figure 2F). Using the NeoTCR gene set scoring method of putative neoantigen-specific T-cell identification [19], NeoTCR4 and NeoTCR8 clusters were annotated (Figure 2G). To track these putative neoantigen-specific T cells in the corresponding TIL drug products (DPs), bulk TCR-seq was performed on the TIL DP. The TCRβ CDR3 clonotypes from the NeoTCR clusters were then identified and quantified in the TIL DP bulk TCR-seq data. Notably, the majority of these putative neoantigen-reactive T-cell clonotypes detected in the tumor digest were preserved in the final TIL product, as demonstrated by proportional and quantitative tracking of TCR clonotypes (Figure 2H–K). While the proportion of neoantigen-specific TCRs varied between the tumor and TIL samples, their consistent presence in expanded products underscores the fidelity of TIL expansion protocols in maintaining tumor-reactive populations. These findings demonstrate that EC-derived TILs retain clinically relevant neoantigen-specific T cells through ex vivo processing, supporting their utility for ACT. Meanwhile, the heterogeneity in clonotype abundance across samples may reflect individual tumor immunogenicity or expansion efficiency, warranting further investigation in a larger population.

### 2.4. Functional Evaluation of Endometrial TIL Reactivity

**Figure 3:**
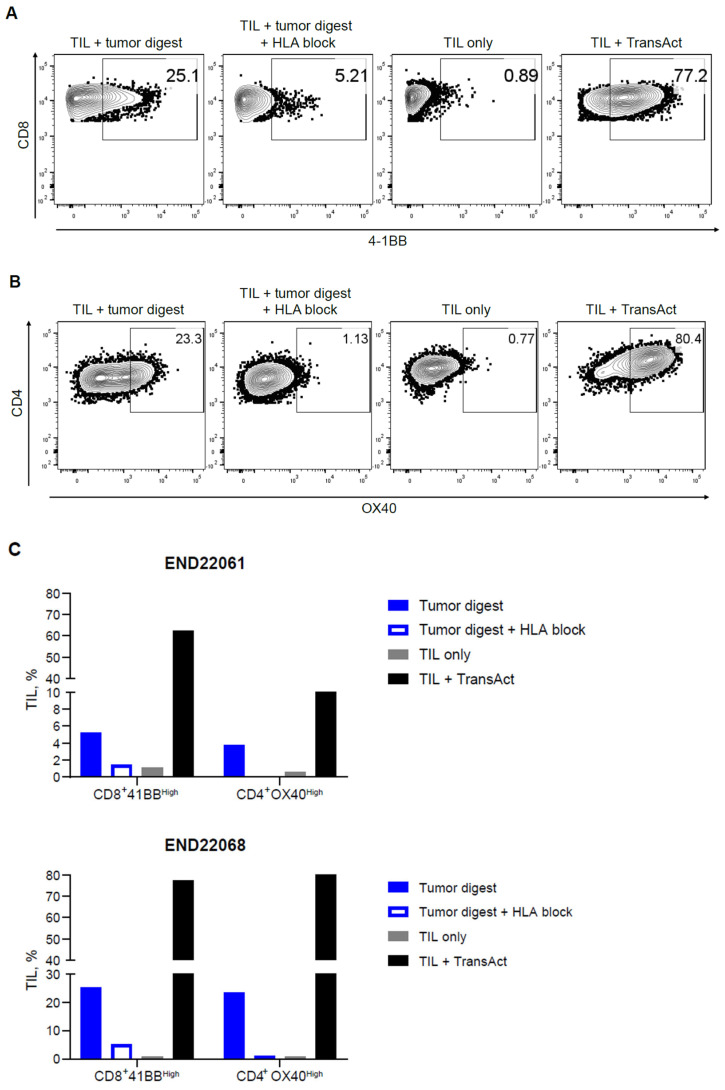
Endometrial drug product TILs are activated when cultured with autologous tumor digest. Endometrial TILs (derived from two donors) were co-cultured with a matched tumor digest with or without HLA-blocking antibodies to block the interaction of TCRs with cognate peptide-MHC tumor antigens. TILs were cultured alone (TILs only) or with TransAct, functioning as negative and positive controls, respectively, for 4-1BB expression in CD8+ TILs, and OX40 expression in CD4+ TILs. (**A**) Representative dot plot for 4-1BB upregulation in CD8+ T cells and (**B**) OX40 upregulation in CD4+ T cells after 24 h of co-culture. (**C**) Summarized data for both TIL donors (END22061 and END22068) demonstrating 4-1BB or OX40 upregulation in an HLA-dependent manner within CD8+ and CD4+ TILs, respectively.

**Figure 4:**
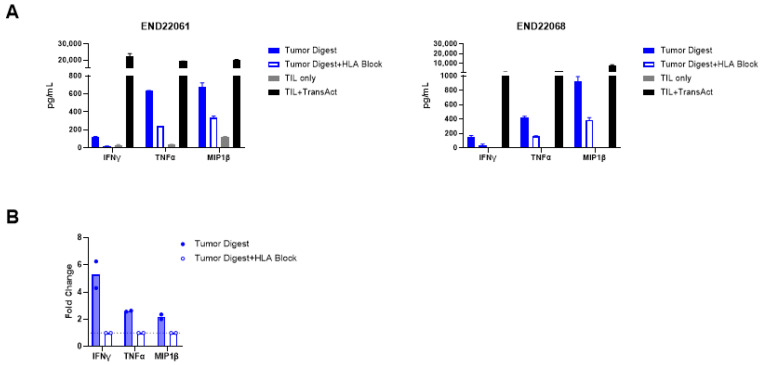
Endometrial drug product TILs produce effector cytokines in response to tumor digest. (**A**) Production of IFN-γ, TNF-α, and MIP-1β by TILs from two donors, END22061 (**left**) and END22068 (**right**), in response to autologous tumor digest stimulation, and the abrogation of cytokine production upon HLA block. TILs were stimulated with TransAct as a positive control, demonstrating robust cytokine production. (**B**) Fold change in cytokine production by TILs cultured with autologous tumor digest in comparison to co-cultures of tumor digest and HLA-blocking antibodies. See also Table S1 for the cytokine values of each patient’s TILs in technical replicates of two.

In addition to identifying putative tumor-reactive TIL clonotypes, the presence of tumor-reactive T cells was confirmed by in vitro co-culture assays. Robust upregulation of 4-1BB within CD8+ TILs and OX40 upregulation within CD4+ TIL upon co-culture with autologous tumor digests were observed. The upregulation of 4-1BB and OX40 was diminished in the TIL-digest co-cultures when HLA blocking antibodies were added, signifying that HLA-peptide recognition by the TIL triggered the expression of 4-1BB and OX40 (Figure 3A–C). Moreover, recognition of autologous tumor digest by TIL induced the production of IFN-γ, TNF-α, and MIP-1β in comparison to TIL cultured without stimulus (TILs only) (Figure 4A). The production of cytokines was 2- to 6.3-fold higher in comparison to cultures with HLA blocking antibodies (Figure 4B; see Table S1). Together, these data establish that expanded EC TIL products retain autologous tumor reactivity in an HLA-dependent manner, with both CD8+ and CD4+ subsets contributing to effector responses through distinct activation pathways (4-1BB vs. OX40) and potent Th1 cytokine production.

### 2.5. In Vitro Antitumor Activity of Endometrial TIL

**Figure 5:**
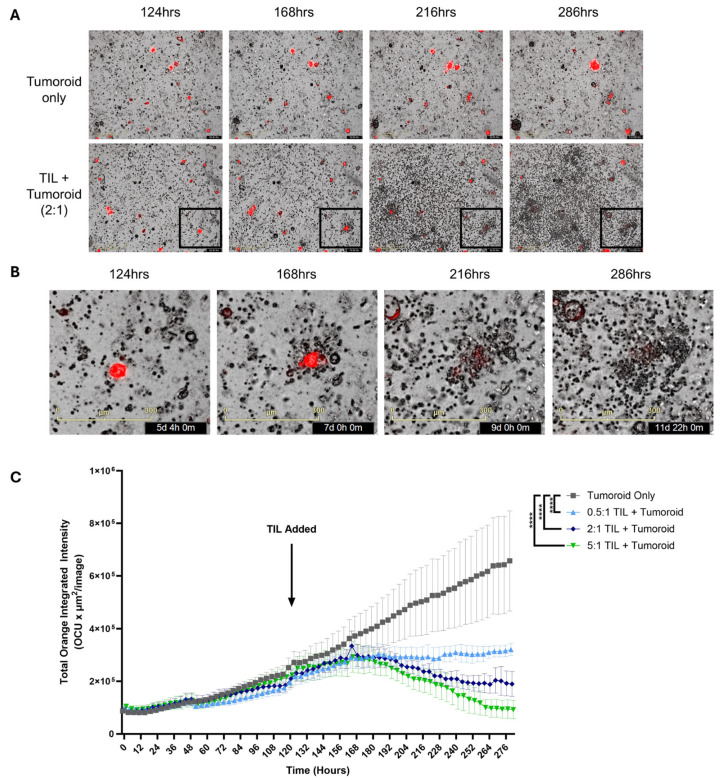
TIL-mediated autologous killing of patient-derived tumoroid. An endometrial tumoroid from a single donor (END22098) stably expressing mCherry was grown for 124 h prior to adding autologous TILs to monitor and assess killing using the Incucyte^®^ system (Sartorius AG, Göttingen, Germany). (**A**) Representative images of the growth of an endometrial tumoroid and its killing of the tumoroid by autologous TILs between 124 and 286 h at 100× magnification. Tumoroid only (top row); TILs+Tumoroid at a 2:1 ET ratio (bottom row). (**B**) Lower right boxed area in A is magnified, demonstrating TIL swarming, tumoroid contact, and the destruction of the tumoroid. (**C**) Incucyte assay comparing tumor cell killing by increasing doses of TILs. Summarized data representing autologous TILs killing of tumoroids, which is dependent on the effector to target (TILs/Tumoroid) ratio. Tumoroids were grown without TILs (gray), 0.5:1 (pale blue), 2:1 (dark blue), and 5:1 (green) TILs/Tumoroid ratios. Data for each group are represented as the mean ± SEM. Statistical analysis was performed using a Friedman test, with Dunn’s multiple comparisons test, **** *p* < 0.0001. Scale bar in (**A**) 400 µm, (**B**) 300 µm.

After identifying that EC TILs can increase the expression of activation markers and produce effector cytokines upon recognition of primary tumor cells within tumor digests, autologous tumor killing in vitro was evaluated. A patient-derived endometrial tumor organoid (tumoroid; END22098) was established. Upon co-culture, the TILs swarmed the tumoroids and mediated the destruction of these cells over multiple days of culture (Figure 5A,B). Moreover, the magnitude of tumor killing was dependent on the effector-to-target (ET) ratio, as demonstrated by strong activity at high ET ratios, which diminished at low ET ratios (Figure 5C). However, the TILs still maintained a level of killing at the lowest ET ratio (0.5:1) conferring a cytostatic effect, preventing the expansion of tumoroids. These data demonstrate that EC TILs can mount robust killing of three-dimensional tumors in vitro over the course of multiple days, recapitulating critical in vivo mechanisms observed in tumor beds required for mediating clinical responses.

### 2.6. Full-Scale Manufacturing of the Endometrial TIL Drug Products

To further validate the manufacturing feasibility, an additional four endometrial tumors from patients were processed at full scale using Gen2 manufacturing close to the clinical manufacturing process. The baseline characteristics of all patients included in this study are presented in Table A1. Full-scale TIL products were successfully achieved for all four EC tumor samples (END22111, END22133, END22134 and END22135), meeting or exceeding critical quality benchmarks (Table 2). All TIL products demonstrated high viability (88.5–95.5%, exceeding the ≥70% target) and purity, with CD45+/CD3+ T-cell enrichment ≥96% (surpassing the ≥90% threshold). The TVC yields ranged from 2.7 × 10^9^ to 77.1 × 10^9^ cells, encompassing the expected target dose range (1 × 10^9^–150 × 10^9^ cells), while tumor cell impurities (TROP2+/EPCAM+) remained minimal (<0.1%). Functional potency, assessed via Dynabead-stimulated IFN-γ release, varied between 3940 and 12,620 pg/mL, reflecting inherent biological variability in TIL effector function. These results confirm the robustness of the manufacturing process across diverse EC samples, consistently generating TIL products with attributes suitable for clinical application. The successful ex vivo expansion of TILs from EC tumors supports the feasibility of TIL manufacturing from EC tumors for use in the adoptive transfer setting.

## 3. Discussion

The current study demonstrates the feasibility of manufacturing TILs from EC tumors using Iovance’s proprietary 22-day Gen2 process at both the research scale and full-scale levels and provides evidence of their antitumor activity. Of the 11 EC tumor samples processed at research scale, 10 (91%) successfully generated a TIL drug product exceeding the target yield of 1 × 10^9^ viable cells, with a median yield of 1.1 × 10^10^ cells and high viability (82.8%). Of the four EC tumor samples processed at full scale, all (100%) achieved the pre-specified TVC and viability targets. These results align with the manufacturing success rates observed in melanoma and other solid tumors, underscoring the robustness of the Gen2 process for TIL expansion in EC.

Neoantigen-driven recognition and T-cell-mediated killing contribute to tumor clearance following ACT with TILs. Accumulating clinical trial data support that the absolute number and frequency of tumor-reactive lymphocytes in TIL drug products from patients with metastatic melanoma highly correlate with clinical efficacy and increased survival [20,21]. In this study, the tumor-reactive lymphocytes in TIL products were assessed using Lowery’s NeoTCR signature [19], confirming the presence of putative tumor-specific TILs in the drug product. The upregulation of activation markers such as 4-1BB on CD8+ T cells [22] and OX40 on CD4+ T cells [23] upon autologous tumor stimulation provides functional evidence of tumor reactivity. These markers are associated with T-cell activation, survival, and memory formation, which are essential for sustained antitumor responses. The increased production of IFN-γ and TNF-α by TILs in response to tumor stimulation highlights their capacity to mount a robust cytokine-mediated anti-tumor response, a hallmark of effective TIL therapy. Furthermore, the killing of tumor cells grown as three-dimensional tumoroids provides evidence that tumor-specific TILs are present in the drug product, supporting their potential to mount cytotoxic activity in vivo. The combination of phenotypic transcriptional, TCR clonotypic, and functional data supports the hypothesis that TIL therapy could be a treatment option for advanced or recurrent EC.

The success of TIL therapy in melanoma has paved the way for its potential application in other solid tumors, including EC. However, several challenges remain. The heterogeneity of EC, both in terms of molecular subtypes and immune infiltration, may influence the efficacy of TIL therapy [14,24]. For example, tumors with high levels of immune cell infiltration, such as those with a POLE mutation or MSI-H status, are hypothesized to be more responsive to TIL therapy and warrant further investigation.

In conclusion, this study demonstrates the feasibility of generating functional TILs from endometrial cancer (EC) using the Gen2 manufacturing process, with robust anti-tumor activity supporting therapeutic potential. The findings support the ongoing phase II trial IOV-END-201 (NCT06481592) evaluating the safety and efficacy of lifileucel in patients with advanced EC. As ACT advances, EC tumor-derived TILs represent a promising strategy to harness the immune system against cancer, offering new hope for patients with advanced or recurrent EC.

## 4. Materials and Methods

### 4.1. Tumor Processing for Expansion of TILs and Immunophenotyping

To evaluate the feasibility of TIL expansion from endometrial carcinomas, fresh tumor specimens weighing more than 1 g were obtained from certified tumor biospecimen providers. All tumor samples were collected under protocols requiring documented informed consent from participating patients. Each tumor sample was accompanied by detailed clinical information, enabling comprehensive analysis. Tumor material was collected irrespective of origin, histologic classification (based on morphology and grade), molecular classification, or prior treatment status. Specifically, patient information contained patient age, race/ethnicity, tumor staging, and molecular profiles. The baseline characteristics of all patients included in this study are presented in Table A1.

The Gen2 TIL manufacturing process used to generate the endometrial TIL final product included a pre-rapid expansion protocol (REP) phase (days 0–11) and REP phase (days 11–22). During pre-REP (days 11–22), performed at 1/10th small-scale and full-scale, 1- to 3 mm tumor fragments were placed in media containing high-dose interleukin-2 (IL-2) for 11 days, allowing TILs to migrate out from the tumor. To further promote TIL growth, TILs were expanded by REP (performed at 1/50th and full-scale) that included irradiated PBMC, IL-2, and anti-CD3 for 11 days. Final harvested REP and in-process samples were assayed for total nucleated cells, TVCs, and viability.

Frozen TILs were thawed and stained with various markers related to exhaustion, activation, memory, and function. Three different panels were used, including a surface staining only panel, an intracellular staining panel using eBioscience™ Foxp3/Transcription Factor Staining Buffer Set (ThermoFisher Scientific, Waltham, MA, USA), and an intracellular staining panel using Monesin and Brefeldin A with anti-CD3 stimulated TILs. Stained cells were analyzed using a BioRad Ze5 flow cytometer (Hercules, CA, USA). Data were analyzed using FlowJo v10.8.1.

### 4.2. TIL Reactivity Assay

REP TILs were labeled with CellTrace Violet (C34557, Invitrogen, Waltham, MA, USA) according to the manufacturer’s instructions. Cryopreserved autologous tumor digest cells (1 × 10^5^) were added to 96-well U bottom plates. Where applicable, HLA-blocking antibodies, Ultra-LEAF^™^ Purified anti-human HLA-A,B,C Antibody (BioLegend, San Diego, CA, USA) and Purified anti-human HLA-DR, DP, DQ Antibody (BioLegend), were added at a final concentration of 10 µg/mL each to tumor digest cultures and incubated for 10 min at room temperature. Then, CellTrace Violet-labeled TILs were added to the culture, followed by an overnight incubation. Each culture consisted of media containing IL-2 (300 IU/mL). For a negative control, TILs were cultured without stimulation (TILs only). For a positive control, TILs were cultured with GMP TransAct (MiltenyiBiotec, Bergisch Gladbach, Germany) according to the manufacturer’s instructions. After overnight culturing, the supernatants were harvested for cytokine measurements of IFN-γ, TNF-α, and MIP-1β. Supernatants were assayed on the Bio-Plex 200 (Bio-Rad, Hercules, CA, USA) using Bio-Plex Pro Human Cytokine IFN-γ Set (171B5019M, Bio-Rad), Bio-Plex Pro Human Cytokine TNF-α Set (171B5026M, Bio-Rad), and Bio-Plex Pro Human Cytokine MIP-1β Set (171B5023M, BioRad). For flow cytometry analysis, TILs were harvested after overnight culturing and labeled with flow cytometry antibodies: CD3 BUV 395 (563546, BD Biosciences, San Jose, CA, USA), CD4 BV 510 (300546, BioLegend), CD8 BV 785 (344740, BioLegend), 4-1BB Alexa Fluor 647 (309824, BioLegend), and OX40 PE-Cy7 (119416, BioLegend). Viability was determined by staining with a LIVE/DEAD™ Fixable Near-IR Dead Cell Stain Kit (L10119, Invitrogen). For analysis, REP TILs were identified by CellTrace Violet staining followed by gating on CD3, CD4, and CD8 to identify upregulation of 4-1BB in CD8+ REP TILs and OX40 in CD4+ REP TILs.

### 4.3. Single-Cell RNA and Bulk TCR Sequencing

Human endometrial tumor material (*n* = 6, biological donors) was digested using the Human Tumor Dissociation kit (Miltenyi PN 130-095-929, Bergisch Gladbach, Germany) and the Miltenyi gentleMACS^™^ Octo Dissociator instrument according to the manufacturer’s protocols. Digested cell suspensions were filtered through 70 µm cell strainers and washed using RPMI medium. CD45+ cells were isolated from the day 0 tumor digest using the StemCell Technologies EasySep^™^ Release Human CD45 Positive Selection kit (PN 100-0105, Vancouver, BC, Canada) and then cryopreserved. Endometrial tumor digest samples (*n* = 6) were obtained from cryopreservation, thawed, and washed twice with PBS + 0.04% BSA. Cell viability was assessed using a Nexcelom Cellaca (Lawrence, MA, USA) automated cell counter with AOPI. Dead cell removal was performed on any sample containing fewer than 75% viable cells using the Miltenyi Dead Cell Removal kit, according to manufacturer guidelines. Single cell Gel-bead emulsions were prepared using 10× Genomics Chromium X controller (Pleasanton, CA, USA). Sequencing libraries were prepared using 10× 5′ Immune profiling v2 reagents following the manufacturer’s guidelines and sequenced on an Illumina NextSeq2000 instrument (San Diego, CA, USA) with an insert read length of 90 base pairs. Primary and secondary data analysis were performed using Cell Ranger (v7.0.0, 10× Genomics) with default parameters. The R package Seurat (v5.0.1) was used for tertiary analysis and data visualization [25]. Genes detected in less than 3 cells were discarded and *TCRA* and *TCRB* family genes were removed to avoid clonotype clustering bias. Dead cells, doublets, or low-quality cells were discarded based on the following empirically determined filter: percent mitochondria reads less than 8% and number of genes detected greater than 500 but less than 6000. Single cell TCR-seq data were added to the single cell meta data, and visualizations were generated using scRepertoire (v1.8.0) [26]. Cells where a TCR was detected and gene expression was consistent with a T cell (e.g., CD3E) were kept for downstream analysis of T-cell subsets. sctransform (v0.4.1) [27] was used for normalization and feature selection, followed by PCA, and integration using harmony (v1.2.0) [28] with default parameters. Clustering was performed at resolution = 2 and UMAP visualizations were generated using Seurat. Automated cell type annotation was performed using scATOMIC (v2.0.2) [29]. For tumor digest T-cell subsets, manual annotation of clusters was performed using cluster marker genes, scATOMIC annotations, and cell-type–specific gene signatures with the R package VISION (v3.0.1) [30]. For gene set analysis, the R packages escape (v1.9.0) [31] and UCell (v2.2.0) [32] were used to quantify the gene set scores for the NeoTCR8 gene signature from Lowrey et al. [19]. TCRβ clonotypes expressed by the NeoTCR8 cluster were identified and exported for further analysis. Heatmap and boxplot visualizations were generated using pheatmap (v1.0.12) [33] and ggplot2 (v3.4.4) [34], respectively.

Matching endometrial TIL drug product samples (*n* = 6) were obtained from cryopreservation, thawed, and washed twice with PBS + 0.04% BSA. Total RNA was extracted from approximately 1 million cells using an NEB Monarch Total RNA Miniprep Kit (Ipswich, MA, USA). Purified total RNA (500 ng) was used as template for preparing TCR beta-chain libraries using Takara SMARTer Human TCR a/b Profiling Kit v2 (Kusatsu, Japan), according to the manufacturer’s guidelines. Equimolar quantities of each library were pooled together and sequenced on a NextSeq2000 instrument (San Diego, CA, USA) with paired end 150 bp reads. High base level sequencing quality of reads was confirmed using DRAGEN’s FastQC module (Illumina DRAGEN v3.10.12). Primary analysis of fastq files to identify and quantify productive rearrangements (clonotypes) was performed using RTCR (v0.5.1) with default parameters [35]. Clonotypes with a less than perfect average base quality score in the CDR3 region were filtered out. Tertiary analysis was performed using the R package immunarch (v0.9.1) [36]. Clonotypes from the NeoTCR8 cluster were identified in the TIL Bulk TCR-Seq and their number and proportion of the total TCR repertoire exported for visualization in GraphPad Prism (v10.0.3).

### 4.4. Tumor Organoid Establishment and TIL Co-Culture Assay

To generate tumoroids, digested cell suspensions were generated as described above and cultured in Cultrex RGF BME Type II, w/o phenol red (R&D systems, Minneapolis, MN, USA, 3536-005-02) in the media listed in Table A3. When a tumoroid grew beyond 2 passages, the cells were transduced with a recombinant lentivirus, pLV[Exp]-Puro-EF1A > mCherry (VectorBuilder, Chicago, IL, USA). mCherry-labeled tumoroids were seeded in 96 well plates for live cell imaging and killing assay using the Incucyte SX5 Live-Cell Analysis imaging system (Sartorius, Göttingen, Germany). After 5 days of growth, autologous TILs were added to tumoroid cultures at various ET ratios. The final media formulation was 50% tumoroid media and 50% TIL media containing IL-2 (300 IU/mL). Tumoroid growth was quantified by the increase in mCherry fluorescence in the orange channel, and killing was quantified by the reduction in mCherry fluorescence over time.

## Figures and Tables

**Table 1:** Summary of product attributes.

Tumor ID	Tumor Size (mg)	Total # of Fragments	TVC (Extrapolation)	Viability (NC200), %	CD45+CD3+%	CD4+CD3+%	CD8+CD3+%
END22061	1083	63	2.71 × 10^10^	90.1	96.6	75.0	23.0
END22062	1100	48	1.24 × 10^10^	82.7	98.1	86.2	12.9
END22063	1032	52	N/A	N/A	N/A	N/A	N/A
END22091	1013	50	6.64 × 10^9^	72.4	98.3	76.2	22.9
END22093	1010	30	8.60 × 10^9^	77.1	97.3	91.7	6.4
END22094	1050	53	1.94 × 10^10^	87.9	90.2	67.0	31.7
END22095	1131	48	8.10 × 10^9^	82.8	97.8	81.6	17.1
END22096	1400	59	9.77 × 10^9^	84.9	93.0	78.8	19.9
END22098	1400	80	3.53 × 10^10^	86.6	97.3	34.4	63.1
END22100	1327	50	3.80 × 10^9^	69.3	95.6	49.4	48.5
END22102	1150	29	1.16 × 10^10^	72.3	94.6	51.3	46.5
**Total, median**			1.07 × 10^10^	82.8	97.0	75.6	23.0

N/A, END22063 did not generate enough viable cells to initiate REP.

**Table 2:** Final product attributes of endometrial TILs manufactured at full scale.

Final Product Attributes	Run 1	Run 2	Run 3	Run 4	Expected Results
	END22111	END22133	END22134	END22135	
Purity (cell viability, %)	88.5	95.5	89.1	89.5	≥70%
Dose (TVC)	5.9 × 10^9^	77.1 × 10^9^	2.7 × 10^9^	68.3 × 10^9^	1 × 10^9^–150 × 10^9^ cells
Identity (% CD45+/CD3+)	96.2	99.4	96.1	99.2	≥90%
Cellular impurity (% TROP2+ or EPCAM+ tumor cells)	0.06	0.04	0.06	0.08	N/A
Potency (Dynabead-based IFN-γ release, pg/mL)	9410	3940	12,620	6475	N/A

N/A, not applicable for these measurement.

## Data Availability

Sequencing data generated during the study have not been made publicly available in a repository due to proprietary considerations and to protect the privacy and confidentiality of the participating patients. Upon reasonable request and authorization by Iovance Biotherapeutics, and after appropriate data-sharing and use agreements have been agreed upon, eligible academic researchers in the field may be provided access to de-identified gene-limited datasets. Requesters should submit a proposal outlining the objective, data format and features, hypothesis and specific rationale to the corresponding authors.

## Supplementary Materials

The following supporting information can be downloaded at https://www.mdpi.com/article/10.3390/ijms26157151/s1.

## Abbreviations

The following abbreviations are used in this manuscript:

ACT	Adoptive cell therapy
ANOVA	Analysis of variance
BSA	Bovine serum albumin
CDR3	Complementarity determining region 3
cDC	Conventional dendritic cell
cDC1	Conventional dendritic cell type 1
cDC2	Conventional dendritic cell type 2
dMMR	Mismatch repair-deficient
DP	Drug product
EC	Endometrial cancer
ET	Effector-to-target
FDA	US Food and Drug Administration
FIGO	International Federation of Gynecology and Obstetrics
Gen2	Generation 2
HLA	Human leukocyte antigen
ICI	Immune checkpoint inhibitor
ICOS	Inducible T-cell costimulator
IFN-γ	Interferon-gamma
IL-2	Interleukin-2
ISG	Interferon-stimulated gene
IU	International units
LAG3	Lymphocyte activation gene-3
LAMP3	Lysosome-associated membrane protein 3
MHC	Major histocompatibility complex
MIP-1β	Macrophage inflammatory protein beta
MMR	Mismatch repair
MSI-H	Microsatellite instability-high
N/A	Not applicable
NeoTCR	Neoantigen-reactive T cells
NIR	Near-infrared spectroscopy
NK	Natural killer
NSCLC	Non-small cell lung cancer
PBMC	Peripheral blood mononuclear cells
PBS	Phosphate-buffered saline
pDC	Plasmacytoid dendritic cell
PD-1	Programmed cell death protein-1
pMMR	Mismatch repair-proficient
pNx	No lymph node was collected
pos	Positive
QC	Quality control
REP	Rapid expansion protocol
scTCR	Single cell T-cell receptor
SD	Standard deviation
SEM	Standard error of the mean
TCR	T-cell receptor
Tem	Effector memory T cells
Tfh	Follicular helper T cells
Th1	T helper 1 cells
TIL	Tumor-infiltrating lymphocytes
TIM3	T-cell immunoglobulin and mucin domain-containing protein 3
TME	Tumor microenvironment
TNF-α	Tumor necrosis factor-alpha
TNM	Tumor, node, and metastasis
Treg	Regulatory T cells
TRM	Tissue-resident memory T cells
TVC	Total viable cells
UMAP	Uniform Manifold Approximation and Projection

## Appendix A

**Table A1:** Clinical and pathological characteristics of all patients are included in this study.

Tumor ID	Tumor Site	Age	Race/ Ethnicity	Pathology	FIGO Grade	TNM Classification	MMR	MLH1 Promoter Methylation
END22061	Uterus	61	White	Endometrioid adenocarcinoma	FIGO grade II	pT3a, pN0	pMMR	
END22062	Uterus	68	White	Endometrioid adenocarcinoma	FIGO grade II	pT1b, pN0	dMMR	81%
END22063	Uterus	67	White	Endometrioid adenocarcinoma	FIGO grade II	pT1b, pN0	dMMR	97%
END22091	Uterus	83	White	Endometrioid carcinoma	FIGO grade II	pT1a, pN0	pMMR	
END22093	Uterus	63	White	Endometrioid carcinoma	FIGO grade II	pT1b, pN0	pMMR	
END22094	Uterus	66	White	Endometrioid adenocarcinoma	FIGO grade I	pT1b, pN0	pMMR	
END22095	Uterus	81	White	Serous carcinoma	High grade (grade III)	pT3a, pN0	pMMR	
END22096	Uterus	45	White	Endometrioid carcinoma	FIGO grade II	pT3b, pNx	dMMR	Positive
END22098	Uterus	67	Hispanic	Endometrioid carcinoma	FIGO grade II	pT1a, pN0	dMMR	Positive
END22100	Uterus	69	White	Endometrioid carcinoma	FIGO grade II	pT1a, pN0	dMMR	MLH1 intact
END22102	Uterus	84	White	Endometrioid carcinoma	FIGO grade III	pT1a, pNx	pMMR	
END22111	Uterus	69	Not available	Endometrioid	FIGO grade I	pT1a, pN0	pMMR	
END22133	Uterus	49	Not available	Endometrioid carcinoma	FIGO grade II	pT1a, pN0	dMMR	MLH1 intact
END22134	Uterus	35	Not available	Endometrioid adenocarcinoma	FIGO grade II	pT1a, pN0	pMMR	
END22135	Uterus	49	White	Adenocarcinoma	Poorly differentiated	T1bN1M0	Not available	

**Table A2:** Count of single cells from EC samples before and after filtering and sub-setting.

Sample	Cell Count Before Filter	Cell Count After Filter	After T-Cell Subset
END22094	6106	5408	1830
END22095	6413	5878	1655
END22096	7722	6612	1705
END22098	12,495	10,544	4761
END22100	5498	4798	732
END22102	4376	3814	667

**Table A3:** Media formulation for endometrial tumoroid.

Media Component	Working Concentration	Manufacturer	Catalog #
17B-Estradiol-Water Soluble	10 mg/mL	Sigma Aldrich (St. Louis, MO, USA)	E4389
A83-01	5 mM	Sigma Aldrich	SML0788-5MG
B27 (50X)	1X	Gibco (Waltham, MA, USA)	17504-044
Chemically defined lipid concentrate	1%	Life technologies (Carlsbad, CA, USA)	11905031
EGF	50 ng/mL	Peprotech (Cranbury, NJ, USA)	AF-100-15
GlutaMAX™ (100X)	1X	Life technologies	35050061
HGF	20 ng/mL	Peprotech	100-39
IGF-1	40 ng/mL	Peprotech	100-11
N2 (100X)	1X	Life technologies	17502001
N-acetyl-cysteine	1.25 mM	Sigma Aldrich	A7250-50G
Nicotinamide	5 mM	Sigma Aldrich	73240-100G
Noggin	10 ng/mL	Peprotech	120-10C
RH R-Spondin 3	25 ng/mL	R&D Systems	3500-RS-025/CF
SB202190	100 nM	Sigma Aldrich	S7067-5MG
Y-27632	10 µM	Selleckchem (Houston, TX, USA)	S1049
Anti-Anti (100X)	1X	Gibco	15-240-062
Advance DMEM/F12	n/a	Invitrogen	12634028

#, number.
