# Supplementary material for: Feasibility of Manufacturing and Antitumor Activity of TIL for Advanced Endometrial Cancers
Source: Int J Mol Sci. 2025 Jul 24;26(15):7151. doi: 10.3390/ijms26157151 (PMC12346291; doi:10.3390/ijms26157151)
Supplement: Supplementary file 1 [file ijms-26-07151-s001.zip › ijms-3742083 - Supplemental Figure S1.pdf]

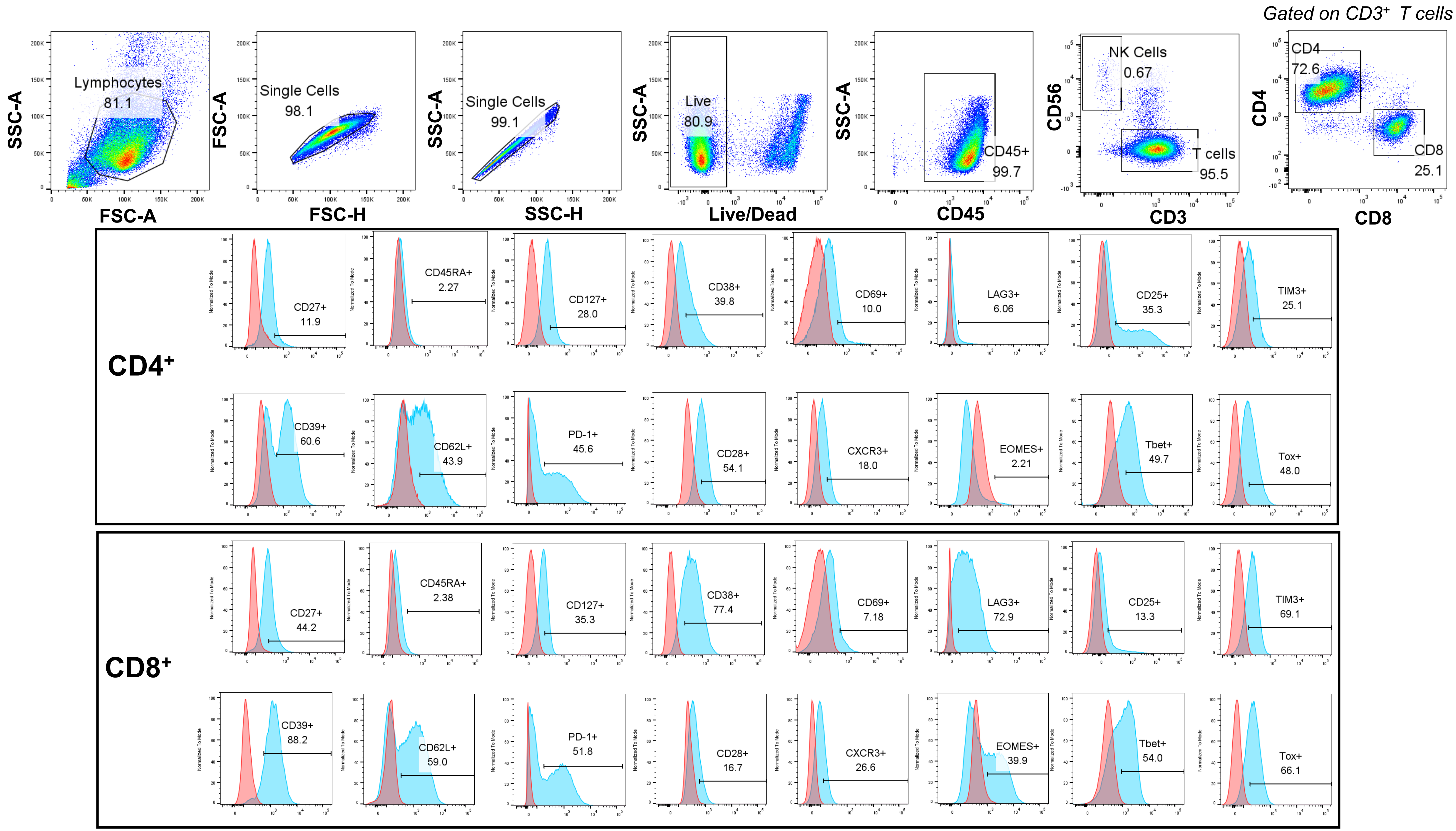

Supplemental Figure S1. Flow cytometry gating strategy related to Figure 1. Sequential gating from left to right is shown, starting from a Lymphocytes gate through the NK cell and T cell lineage gate. CD4 and CD8 T cells were gated on CD3+CD56- cells. Surface markers and transcription factors gated on $CD4^{+}$ or $CD8^{+}$ T cells are represented as histograms normalized to Mode. Red, FMO control for surface markers; Isotype control for transcription factors. The percent positivity of each marker is shown on each graph corresponding to the blue histograms.
