# Supplementary material for: Feasibility of Manufacturing and Antitumor Activity of TIL for Advanced Endometrial Cancers
Source: Int J Mol Sci. 2025 Jul 24;26(15):7151. doi: 10.3390/ijms26157151 (PMC12346291; doi:10.3390/ijms26157151)
Supplement: Supplementary file 1 [file ijms-26-07151-s001.zip › ijms-3742083 - Supplemental Table S1.pdf]

Table S1. Raw data for cytokine measurements generated from the Bio-Plex 200. Refers to Figure 4.

Sample	Cytokine	Tumor Digest			Tumor Digest+HLA Block			TIL only			TIL+TransAct			Fold Change vs HLA Block
		Mean (pg/mL)	*SD*	*Replicates*	*Mean (pg/mL)*	*SD*	*Replicates*	*Mean (pg/mL)*	*SD*	*Replicates*	*Mean (pg/mL)*	*SD*	*Replicates*	
END22061	IFN-γ	120.96	5.09	2	19.32	2.61	2	28.51	7.78	2	22083.12	1915.8	2	6.26
	TNF-α	633.4	4.29	2	239.52	1.44	2	37.48	1.49	2	19360.86	88.41	2	2.64
	MIP-1β	679.79	41.71	2	337.2	16.24	2	125.37	0.68	2	20321.68	84.92	2	2.02
END22068	IFN-γ	150.61	20.3	2	34.92	18.12	2	0	0	2	1159.43	101.31	2	4.31
	TNF-α	423.68	15.79	2	164.8	4.35	2	0	0	2	1705.69	96.2	2	2.57
	MIP-1β	917.42	72.95	2	388.6	33.06	2	0	0	2	8069.87	571.78	2	2.36

HLA, human leukocyte antigen; IFN-γ, interferon-gamma; MIP-1β, macrophage inflammatory protein beta; SD, standard deviation; TIL, tumor-infiltrating leukocytes; TNF-α, tumor necrosis factor-alpha.
